# Positive Childhood Experiences and the Indirect Relationship With Improved Emotion Regulation in Adults With ADHD Through Social Support

**DOI:** 10.1177/10870547241261826

**Published:** 2024-06-24

**Authors:** Catherine T. Lowe, Alexandra C. Bath, Brandy L. Callahan, Emma A. Climie

**Affiliations:** 1 University of Calgary, AB, Canada

**Keywords:** emotion regulation, ADHD, positive childhood experiences, social support

## Abstract

Individuals with ADHD report diminished emotion regulation capacities and adversity in childhood detrimentally affects emotion regulation development; however, how positive childhood experiences (PCEs) and whether and how social support are related to PCEs and emotion regulation for those with ADHD is unknown.

**Objective:**

To identify direct and indirect associations between PCEs and social support to emotion regulation outcomes in adults with ADHD.

**Method:**

Adults with ADHD (*n* = 81) reported PCEs, current social support, and emotion regulation. Conditional effects modeling examined the direct and indirect relationships between PCEs and emotion dysregulation through social support.

**Results:**

Higher PCEs were indirectly related to improved emotion regulation through increased social support generally (β = −.70, 95% CI [−1.32, −0.17], and specifically through belonging (β = −.43, 95% CI [ −0.87, −0.05], self-esteem (β = −.61, 95% CI [−1.08, −0.27], and tangible social support (β = −.50, 95% CI [−1.07, −0.02].

**Conclusions:**

PCEs may protect emotion regulation in adults with ADHD through social support, possibly through facilitating social connections, increasing access to social support, and sustaining emotion regulation strategies.

Attention-deficit/hyperactivity disorder (ADHD) is a neurodevelopmental disorder characterized by a persistent pattern of inattention, hyperactivity, or impulsivity that interferes with functioning or development, affecting 2.5% to 4.4% of the adult population, and up to 26.7% of outpatient psychiatric patients (Adamis et al., 2022; American Psychiatric Association, 2022; Faraone et al., 2021; Kessler et al., 2006). In addition to diagnostic symptoms, those with ADHD often struggle with emotion regulation (Shaw et al., 2014). Additionally, it has been suggested that deficits in regulation are a primary characteristic of ADHD (Barkley, 2020; Shaw et al., 2014; Soler-Gutiérrez et al., 2023). Therefore, understanding factors that influence emotion regulation development is important to identify means to improve the overall health and well-being of individuals with ADHD.

Emotion regulation encompasses the capacity to alter one’s emotional state to support goal-oriented, adaptive behaviors (Gratz & Roemer, 2004; Naragon-Gainey et al., 2017; Sheppes et al., 2015). Appropriate emotion regulation capacities allow individuals to effectively identify, select, and implement strategies such as cognitive reappraisal when confronted with varying contextual emotional, cognitive, and motivational factors (Naragon-Gainey et al., 2017; Sheppes et al., 2015). However, there are differences in preferences and perceptions of efficacy among these strategies, which results in varying degrees of emotion regulation (Gratz & Roemer, 2004; Gross & John, 2003).

Poor emotion regulation comprises maladaptive strategies that interfere with goal attainment (Aldao & Nolen-Hoeksema, 2012). For example, problems with attention or behavioral avoidance, being overly preoccupied with or rejecting unpleasant feelings, anxiety, self-destructive activities, maladaptive thoughts, behavioral challenges, emotional suppression, or unpredictable emotional reactions are all instances of emotion dysregulation exhibited by those with ADHD (Naragon-Gainey et al., 2017). Furthermore, emotion dysregulation has detrimental consequences beyond the inability to cope with problems and disrupting goal achievement. For example, over time, the use of poor emotion regulation strategies can become habitual (Mauss et al., 2007), resulting in ongoing maladaptive emotion dysregulation and consequently resulting in poorer mental health (Andrews et al., 2021), less social support (Gross & John, 2003), and higher levels of emotional distress (Robson et al., 2020), demonstrating the adverse outcomes and functional impacts for those with emotion regulation challenges.

Consistently, individuals with ADHD experience increased rates of emotion regulation challenges marked with functional impairments relative to their neurotypical peers (Cherkasova et al., 2022). Considerably problematic, emotion dysregulation occurs throughout the lifespan and may even be a factor in the relationship between ADHD diagnosis and impairments in critical functional domains, internalizing symptoms, and relationship satisfaction (Bodalski et al., 2019). Overall, adults with dysfunctional emotion regulation and ADHD face exacerbated challenges relative to those with adequate emotion regulation capacities and consequently have decreased daily life functioning, lower quality of life, and poorer mental health (Ben-Dor Cohen et al., 2021; Bodalski et al., 2019), demonstrating the critical need to understand determinants of emotion regulation development in order to develop effective interventions.

One critical component in the development of effective emotion regulation capacities potentially stems from early childhood experiences. Early childhood experiences, for better or for worse, affect functioning into adulthood (Masten & Barnes, 2018; Rollins & Crandall, 2021). For instance, adversity in developmental periods can have later impairing impacts on health and well-being. One such example of adversity includes adverse childhood experiences (ACEs), which are traumatic events that occur before the age of 18 years that may involve maltreatment (neglect, or physical, emotional, or sexual abuse), parental loss or parental maladjustment (Felitti et al., 1998), and increase risk for psychological and emotional regulation challenges in adulthood (Felitti et al., 1998; Gruhn & Compas, 2020; Merrick et al., 2017; Rudenstine et al., 2019). Largely, ACEs have demonstrated associations with poor outcomes in health and well-being (Gruhn & Compas, 2020). Conversely, positive childhood experiences (PCEs) are associated with improved health and functional outcomes (Bethell et al., 2019). PCEs involve the child, parent, and parent-child relationship, and contribute to achieving optimal child health outcomes in the physical, cognitive, social, and emotional developmental domains (Sege & Harper Browne, 2017). PCEs encompass social support, nurturing relationships, and secure attachment with parents and other supportive adults that can foster resilience and well-being in adulthood (Cohen & Wills, 1985; Crandall et al., 2019; Masten & Barnes, 2018; Narayan et al., 2021), influencing positive health overall and lasting well-being (Bethell et al., 2019).

There is a robust body of evidence that increased ACE exposures are related to poorer emotion regulation generally (Weissman et al., 2019), and individuals with ADHD are at an increased risk of experiencing ACEs (Lugo-Candelas et al., 2021), potentially compounding already heightened emotion regulation deficits in this population (Ben-Dor Cohen et al., 2021; Bodalski et al., 2019). In contrast, literature assessing the relationship between emotion regulation and PCEs is notably absent overall, particularly in the ADHD population, and demands future research attention. Consequently, how PCEs affect emotion regulation in individuals with ADHD and whether other protective factors impact emotion regulation development is unknown.

One powerful predictor protective for health and well-being is social support (Cohen & Wills, 1985; Cohen, 2004; Feeney & Collins, 2014; Holt-Lunstad et al., 2017; Tomfohr et al., 2015). Social support encapsulates the means that social connections facilitate health and well-being through the perceived access to emotional, informational, or instrumental social resources in personal relationships (Cohen et al., 2001; Cohen, 2004; Feeney & Collins, 2014). Moreover, social support is related to improved utilization of effective emotion regulation strategies (Marroquín & Nolen-Hoeksema, 2015) and demonstrates protective features for emotional well-being both in the general population (Cohen & Wills, 1985; Holt-Lunstad et al., 2017) and for those with ADHD specifically (Connolly et al., 2023; Mastoras et al., 2018). In terms of the relationship between social support and emotion regulation, this potential association can be viewed through the perspective of social support in circumstances of perceived stress. According to stress theory, experiencing situations in which environmental demands exceed the coping capacities of the individual causes harmful stress with consequences to health and well-being; however, moderators of stress, such as social support, facilitate adequate coping strategies to buffer and mitigate threats, resulting in improved overall health and well-being (Schneiderman et al., 2005). Therefore, it is reasonable to hypothesize that social support may also preserve or even facilitate emotion regulation.

In fact, one avenue through which individuals attempt to regulate emotion is through interpersonal emotion regulation (IER) strategies, where individuals attempt to cope with stress and subsequent emotional experiences through support-seeking behaviors (Zaki & Williams, 2013). Within the IER framework, social support is an essential component of emotion regulation in which social connections are a critical resource for utilizing effective strategies through access to social support to cope with various contexts (Zaki & Williams, 2013). Furthermore, how social support is perceived also affects well-being, and individuals report improvements when they perceive the support as beneficial in regulating their emotions (E. O. Cheung et al., 2015; Cohen et al., 1986).

There are different types of social support that an individual can access, including appraisal support, self-esteem support, belonging support, and tangible support (Cohen & Hoberman, 1983; Cohen & Wills, 1985). Appraisal support involves receiving others’ feedback and guidance, which helps them evaluate situations, clarify goals, and plan actions (Cohen & Wills, 1985). Self-esteem support involves self-expression to boost self-worth and self-efficacy, helping individuals overcome self-doubt, cope with failure, and pursue aspirations (Cohen & Wills, 1985). Belonging support provides security, acceptance, and identity; when an individual feels supported by others, they feel connected with others who share interests, values, or experiences (Cohen & Wills, 1985). Finally, tangible support involves taking on responsibilities to help others manage their problems (Cohen & Wills, 1985).

Clearly, social support protects health and well-being and potentially facilitates effective emotion regulation strategies. However, individuals with ADHD often report difficulty accessing social support (Bernardi et al., 2012; Mastoras et al., 2018). For example, two studies looked at levels of social support in children and youth with ADHD compared to normative samples and found that children with ADHD reported less social support than their neurotypical peers (Harris-Lane et al., 2021; Mastoras et al., 2018). Comparatively, in the adult population, three studies have looked at levels of social support reported by adults with ADHD compared to neurotypical individuals and found mixed results. Consistent with the child studies on ADHD (Harris-Lane et al., 2021; Mastoras et al., 2018), one study found that adults with ADHD reported less social support (Bernardi et al., 2012). However, alternative studies determined that overall social support did not differ between those with and without ADHD diagnosis (Alvarez-Fernandez et al., 2017; Connolly et al., 2023).

At the time of writing, no studies evaluated the role of social support on emotion regulation for individuals with ADHD; however, identifying social support as a powerful protective agent in well-being, some studies assessed how social support affected mental health outcomes in those with and without ADHD. Noting the relationship between mental well-being and emotion regulation, it is reasonable to suggest that social support may relate similarly to the outcomes observed. For example, one study evaluated how social support and ADHD diagnosis were associated with anxiety and depressive symptoms in adults (Connolly et al., 2023). Although this study did not evaluate emotion regulation directly, it did demonstrate robust results for social support generally. Specifically, higher social support in adulthood was associated with lower anxiety and depressive symptoms; however, there were no differences between those with and without ADHD, demonstrating the universal protective impact of social support (Connolly et al., 2023). Additionally, another study evaluated the relationship of perceived social support between neurotypical adults, those with autism spectrum disorder (ASD), and adults with ADHD (Alvarez-Fernandez et al., 2017). Alvarez-Fernandez et al. (2017) identified significantly lower levels of social support reported between those with ASD and the other groups in specific domains of social support; however, no differences were identified between those with and without ADHD, suggesting that contrary to alternative studies (Bernardi et al., 2012; Harris-Lane et al., 2021; Mastoras et al., 2018), access to social support was no different for those with and without ADHD. Clearly, the literature remains mixed and requires further exploration.

Given that social support is generally protective for emotional well-being, it is reasonable to suggest that it may also be protective for emotion regulation for those with ADHD. However, how childhood experiences influence obstacles to social support and are related to emotion dysregulation is unknown and necessary to address to improve long-term emotion regulation in individuals with ADHD.

Social support and PCEs demonstrate robust relationships with improved well-being generally and may facilitate improved coping in times of stress, demonstrating good emotion regulation. However, for individuals with ADHD, who experience worsened emotion dysregulation relative to neurotypical individuals, how PCEs and social support interact and are related to emotion regulation is unknown and critical to understanding in order to generate effective targeted interventions. Only two studies evaluated childhood experiences and social support and assessed mental well-being, while no studies to date have assessed PCEs, social support, and emotion regulation in adults with ADHD. One source of stress that affects emotion regulation is adversity in childhood. In the context of ACEs, a significant source of stress, social support protects and buffers its impact, preserving well-being (Lee et al., 2022). In contrast, concurrent with PCEs, social support may positively affect mental well-being (Bethell et al., 2019; Schofield et al., 2013); however, how these impact emotion regulation generally or in those with ADHD is unknown. Taken together, these studies suggest that childhood experiences and social support may interact and independently be associated with well-being and potentially emotion regulation generally; however, how this affects those with ADHD is unknown.

Although research has identified protective factors for well-being, such as social support and PCEs, research for long-term adult outcomes has been limited; studies have mixed findings (Alvarez-Fernandez et al., 2017; Bernardi et al., 2012; Connolly et al., 2023), do not assess groups with ADHD (Bethell et al., 2019; Cohen & Wills, 1985; Crandall et al., 2019; Masten & Barnes, 2018; Narayan et al., 2021), focus solely on youth with ADHD (Harris-Lane et al., 2021; Mastoras et al., 2018), or evaluate either social support or childhood experiences individually (E. O. Cheung et al., 2015; Cohen, 2004; Cohen et al., 1986; Feeney & Collins, 2014; Holt-Lunstad et al., 2017; Schneiderman et al., 2005; Tomfohr et al., 2015). Notably, few studies assess emotion regulation directly (Marroquín & Nolen-Hoeksema, 2015; Zaki & Williams, 2013), and no studies to date assess how PCEs and social support interact and relate to emotion regulation in adults with ADHD. One manner in which PCEs may be associated with better emotion regulation outcomes is through facilitating social connections and social integration in social networks, providing increased access to social support, and, in turn, improved emotion regulation capacities.

## Current Study

It is clear that those with ADHD may experience exacerbated or increased emotion regulation dysfunction, and that emotion regulation is associated with childhood experiences. Additionally, social support is potentially protective for improved emotion regulation in the general population. However, no studies have assessed the relationship between PCEs and emotion regulation in adults with ADHD, nor how social support may mediate associations between PCEs and subsequent emotion regulation capacities for adults with ADHD. The current study assessed associations between PCEs and social support with later adult emotion regulation capacities in individuals with ADHD and conditional effects relationships between childhood PCEs and adult emotion regulation capacities through social support. It was hypothesized that both increased PCEs and higher reports of social support were independently associated with better emotion regulation in adults with ADHD, regardless of the type of social support offered. Furthermore, it was expected that social support mediated the relationship between PCEs and subsequent emotion regulation in adults with ADHD, such that PCEs promote social connections and integration, facilitating access to social supports, and are therefore related to better emotion regulation outcomes.

## Methods

The present study was a sub-study utilizing data from a larger longitudinal project assessing relationships between major life events and ADHD symptoms; only a subset of measures collected at study entry are included in the present study. All participants provided informed consent and received an honorarium via gift card for participation. All procedures were approved by the host institution’s Research Ethics Board (#20-1103).

## Participants

Participants were recruited using convenience and snowball sampling techniques from September 2021 through September 2023. Advertisements for recruitment were posted online through social media, community groups, a university-based research participation website, and a registry of participants held by the primary researcher. Flyers were also distributed for recruitment to community centers, events, and ADHD clinical sites in the local community. To participate in the current study, individuals must have been fluent in English and had normal or corrected-to-normal hearing and vision abilities. Exclusion criteria consisted of a history of stroke or cognitive impairment as determined by a score of less than 22 on the Telephone Interview for Cognitive Status (TICS; Brandt et al., 1988). Of 129 participants recruited to participate in the larger study, 105 provided informed consent and went on to complete all study measures. Only those with a formal diagnosis of ADHD, ascertained by self-report (*n* = 67) or by clinical interview, described below (*n* = 14), and had fewer than two missing data points were included in the current sample for analyses for a total of 81 participants.

## Procedure

During a telephone interview with a trained research assistant, participants completed the TICS (Brandt et al., 1988), and those who had not been formally assessed for ADHD by a healthcare professional also completed the Structured Clinical Interview for DSM-5 (SCID-5; First, 2015), with all participants exceeding the clinical threshold for ADHD using the Conners Adult ADHD Rating Scales (CAARS; Conners et al., 1999). None were excluded on the basis of their TICS score. Following the telephone assessment, participants received a personalized email link to complete online questionnaires for all study information. Participants provided demographic information and answered questions about their childhood experiences, current social support, and emotion regulation capacities.

## Measures

### Demographic Information

Demographic information was collected from all participants, including their age (years), ethnicity, gender, and current ADHD medication use. Due to the disproportionate number of participants indicating their ethnicity was White, ethnicity was coded as (1) White and (2) not White. Similarly, because education was highly skewed, self-reported education experience was identified as having completed post-secondary training or below (1) or having any form of post-secondary education (2).

### Childhood Experiences

Participants completed a questionnaire asking them to indicate whether they had experienced specific PCEs shown to be protective for developmental outcomes, based on a standard measure developed through a 2015 population study (Bethell et al., 2019). Participants indicated how frequently they had experienced each of seven PCEs through a 5-point Likert scale from “Never” to “All the Time.” Scores were summed to a maximum of 35, with higher scores indicating increased reports of PCEs. PCEs were internally reliable across all questions with Cronbach’s α = .79.

### Social Support

Current social support was assessed using the Interpersonal Support Evaluation List (ISEL; Cohen & Hoberman, 1983), a reliable and validated scale in which participants ranked perceived measures of support using a 4-point Likert scale ranging from “Definitely True” to “Definitely False.” Responses were summed to a maximum potential of 120, in which higher scores indicated higher perceived social support. The ISEL further assesses social support through four subscales to represent tangible support, belonging support, self-esteem support, and appraisal support. The measure was internally reliable, with Cronbach’s α = .83, across subscales and total social support scores.

### Emotion Dysregulation

Emotion dysregulation was assessed using the Difficulties in Emotion Regulation Scale (DERS), a validated clinical scale to assess problems in emotion regulation (Gratz & Roemer, 2004, 2008; Hallion et al., 2018). This scale is a 36-item assessment that uses a 5-point Likert scale for participants to indicate the frequency with which statements regarding emotion regulation are true for them, from “Almost Never” to “Almost Always.” The DERS assesses six sub-types of emotion regulation capacities, including non-acceptance of emotional responses, difficulty in engaging in goal-directed behavior, impulse control difficulties, lack of emotional awareness, limited access to emotion regulation strategies, lack of emotional clarity, and an overall rating of problems with emotion regulation. Scores are summed to assess each subscale in emotion regulation problems, and the total sum across subscales represents the overall score in emotion regulation problems. Higher scores on the DERS indicate increased emotion regulation difficulties. Internal reliability for the DERS was good, with Cronbach’s α = .80 across each sub-scale and total emotion dysregulation score.

## Analytic Approach

All variables were examined for impossible values and extreme outliers, and none were removed. Some data were missing, as reported in Tables 1 and 2, with each missing variable under 5% absent. Data were missing completely at random based on Little’s MCAR test (χ^2^_*(13)*_ = 12.06, *p* = .523), and multiple imputations with five iterations were used to input missing responses for analyses. Data cleaning and bivariate correlations were completed using IBM SPSS Statistics for Windows (Version 28).

**Table 1.** Sample Characteristics (*n* = 81).

Variable	Mean ± *SD* or % (*n*)	Median (range)	Missing data % (*n*)
Age (years)	36.79 ± 14.61	34 (18–79)	4.9 (4)
Ethnicity			
White	82.7 % (67)		
African Canadian/Black	2.5 % (2)		
Indigenous (First Nations, Metis, Inuit)	2.5% (2)		
East Asian	1.2% (1)		
Southeast Asian	1.2% (1)		
South Asian	3.7% (3)		
Latin, Central, and South American	2.5% (2)		
Middle Eastern	1.2% (1)		
Prefer not to say	1.2% (1)		
Other	1.2% (1)		
Education			3.7 (3)
Completed middle school (grade 9)	2.5% (2)	—	
Completed high school (grade 12 or 13)	22.2% (18)	—	
Completed a college/trade school/CEGEP (2-year post-secondary degree)	19.8% (16)	—	
Completed a bachelor’s degree (3- or 4-year undergraduate degree)	37.0% (30)	—	
Completed a master’s degree	14.8% (12)	—	
Gender			3.7 (3)
Female	75.3% (61)	—	
Male	18.5% (15)	—	
Other	2.5% (2)	—	
Current medication use			
Currently using ADHD medication	40.7% (33)		
No current ADHD medication use	59.3% (48)		

**Table 2.** Descriptive Data for Study Variables of Interest.

Variable	Mean ± *SD*	Median (range)	Missing data % (*n)*
Emotion regulation problems (DERS)	89.65 ± 18.95	90.0 (40–132)	1.2 (1)
Social support	74.95 ± 24.62	79 (15–114)	2.5 (2)
Appraisal social support	19.90 ± 8.35	22 (0–30)	2.5 (2)
Tangible social support	20.86 ± 7.01	22 (2–30)	2.5 (2)
Self-esteem social support	16.44 ± 5.63	17 (2–27)	2.5 (2)
Belonging social support	17.67 ± 6.86	17 (4–29)	2.5 (2)
Positive childhood experiences (PCEs)	20.73 ± 5.50	21 (8–35)	3.7 (3)

Five bias-corrected models based on 2,000 bootstrapped samples were used to identify conditional effects modeling analyses to assess the direct and indirect effects of PCEs on emotion dysregulation through social support broadly, as well as through each sub-type of social support using R version 4.1.2 statistical software (R Core Team, 2021) and the MeMoBootR and manymome packages (Buchanan, 2018; S. F. Cheung & Cheung, 2023).

## Results

### Descriptive Statistics

Sample characteristics are reported in Table 1. On average, participants were 38 years old, ranging from 18 to 79 years of age and were predominantly female (75%) and White (83%). More than 96% of participants had completed at least high school education, with 72% having completed post-secondary training. At the time of study, 40% of participants were accessing medication for treatment of their ADHD,

Descriptive data for all study variables are displayed in Table 2. Participants reported an average emotion dysregulation score (*m* = 89.7) similar to those presenting for outpatient treatment with at least one DSM disorder (Gratz & Roemer, 2004). Moderate levels of social support overall were reported (*m* = 75), with the highest scores on tangible (*m* = 20.9) and appraisal (*m* = 19.9) social support and the lowest on self-esteem social support (*m* = 16.4). Participants indicated moderate PCE scores (*m* = 20.7), with some participants reporting infrequent positive experiences in childhood (8), while others reported they had experienced all PCEs to their fullest extent (35), representing a diverse range of childhood experiences.

Bivariate correlations for all variables are displayed in Table 3, which indicate that participants’ age was related to ethnicity (*r* = −.37, *p* < .001) and gender (*r* = −.30, *p* = .006), and that gender was further related to total emotional regulation difficulties (*r* = −.24, *p* = .031), such that men reported increased problems with emotion regulation. Additionally, that participant ethnicity was related to current ADHD medication use (*r* = .33, *p* = .003), with White participants indicating lower current medication use. Current medication use was not associated with emotion regulation (*r* = .19, *p* = .088), however, was correlated with social support broadly (*r* = −.24, *p* = .029), as well as appraisal (*r* = −.25, *p* = .024), tangible (*r* = −.23, *p* = .043), and belonging social support specifically (*r* = −.25, *p* = .024). Total social support was correlated with all variables of interest. Specifically, higher levels of social support overall were related to lower scores of emotion regulation difficulties (*r* = −.48, *p* < .001), with higher scores on each subtype of social support showing significant relationships with improved emotion regulation abilities for each appraisal (*r* = −.36, *p* ≤ .001), tangible (*r* = −.41, *p* ≤ .001), self-esteem (*r* = −.54, *p* < .001), and belonging social support (*r* = −.43, *p* ≤ .001) when assessed individually. Higher reports of PCEs were also related to better emotion regulation abilities (*r* = −.34, *p* = .002). Moreover, increased reports of PCEs were associated with higher incidences of social support overall (*r* = .53, *p* < .001) and with each subtype of social support: appraisal (*r* = .51, *p* < .001), tangible (*r* = .51, *p* < .001), self-esteem (*r* = .39, *p* < .001), and belonging (*r* = .45, *p* < .001).

**Table 3.** Correlations Among Key Study Variables and Sociodemographic Characteristics of the Sample.

Variable	1.	2.	3.	4.	5.	6.	7.	8.	9.	10.	11.
1. Age	—	−.30**	−.01	−.37**	−.13	−.01	−.17	−.05	−.12	−.11	.11
2. Gender		—	−.17	−.18	.18	−.24*	.22	.16	.10	.15	−.15
3. Med use			—	.33**	−.24*	.19	−.25*	−.23*	−.10	−.25*	−.07
4. Ethnicity				—	−.09	.01	−.11	−.19	.03	−.02	.12
5. Total social support					—	−.48**	−.93**	.90**	.78**	.90**	.53**
6. Total emotion dysregulation						—	−.36**	−.41**	−.54**	−.43**	−.34**
7. Appraisal social support							—	.81**	.61**	.79**	.51**
8. Tangible social support								—	.61**	.73**	.51**
9. Self-esteem social support									—	.62**	.39**
10. Belonging social support										—	.45**
11. PCEs											—

* *p* < .05. **p < .001.

## Conditional Effects Modeling

**Figure 1.**
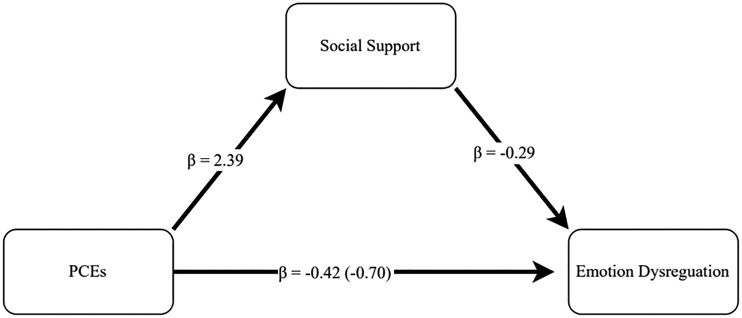
Conditional effect modeling assessing the direct and indirect effects of PCEs on emotion regulation through social support. *Note.* PCEs were indirectly related to emotion dysregulation through social support (β = −1.18) when controlling for all covariates and variables of interest at every level of the model.

Total Social Support. Conditional effects modeling using bias-corrected intervals on 2,000 bootstrapped samples indicated that although PCEs were not directly associated with adult emotion regulation (β = −.42, *p* = .287), that they were indirectly related to improved emotion regulation through social support (β = −.70, 95% CI [−1.32, −0.17]. Specifically, higher reported PCEs were associated with increased social support (β = 2.39, *p* < .001), which, in turn, was related to improved emotion regulation capacities (β = −.29, *p* = .004), as seen in Figure 1.

## Subtypes of Social Support

Additional models were generated to assess the effects of PCEs on emotion regulation through each appraisal, tangible, self-esteem, and belonging subtype of social support while controlling for all subtypes and covariates to better understand this complex relationship. Using a bias-corrected 2,000 bootstrapped sampling procedure, each model demonstrated unique effects explaining the variance observed in emotion regulation.

### Appraisal Social Support

PCEs were neither directly (β = −.80, *p* = .055) nor indirectly (β = −.32, 95% CI [−0.88, 0.16]) related to emotion regulation. Similarly, although higher PCEs were associated with increased appraisal social support (β = .79, *p* < .001), appraisal social support showed no relationship with emotion regulation (β = −.41, *p* = .188).

### Tangible Social Support

Increased PCEs were directly associated with higher tangible social support (β = .65, *p* < .001), which was in turn related to improved emotion regulation (β = −.77, *p* = .031), but PCEs had no direct effects on emotion regulation outcomes (β = −.62, *p* = .121). However, higher PCEs were associated with improved emotion regulation through higher tangible social support (β = −.50, 95% CI [−1.07, −0.02]).

### Self-Esteem Social Support

PCEs were also indirectly associated with improved emotion regulation through self-esteem social support (β = −.61, 95% CI [−1.08, −0.27]). Specifically, that more reported PCEs were associated with increased self-esteem social support (β = .40, *p* < .001), which was then related to improved emotion regulation outcomes in adults with ADHD (β = −1.54, *p* < .001), even though PCEs had no direct effects (β = −.51, *p* = .122).

### Belonging Social Support

PCEs showed no direct relationship with emotion regulation outcomes (β = −.68, *p* = .065), while increased belonging social support was associated with improved emotion regulation (β = −.80, *p* = .017). However, higher PCEs were related to increased belonging social support (β = .54, *p* < .001), and were indirectly associated with improved emotion regulation through higher belonging social support (β = −.43, 95% CI [−0.87, −0.05]).

## Discussion

The current study assessed the direct and indirect relationships between PCEs, social support, and emotion dysregulation in adults with ADHD using conditional effects modeling. Similar to existing research, in which social support is associated with improved health and well-being generally (Marroquín & Nolen-Hoeksema, 2015) and in those with ADHD specifically (Bernardi et al., 2012; Connolly et al., 2023; Harris-Lane et al., 2021; Mastoras et al., 2018), the current study demonstrated an independent relationship with emotion dysregulation, such that higher overall social support was related to improved emotion regulation. Although not directly predictive or related to emotion regulation outcomes, interestingly, higher reported PCEs were indirectly associated with improved emotion regulation through increased social support broadly, but also when assessing each individual type of social support, suggesting that childhood experiences may affect the ability to access social support. One possible reason for this association is that PCEs may facilitate the expansion of the social network, increasing social integration and resulting in improved access to social support (Crandall et al., 2019; Kosterman et al., 2011; Sege & Harper Brown, 2017).

However, although social support, both broadly and all specific subtypes mediated the relationship between PCEs and emotion dysregulation, direct relationships were non-significant, suggesting that how PCEs affect social supports and later emotion dysregulation in adults with ADHD is more nuanced than first hypothesized. One possible explanation for the absence of a significant pathway is that social support is derived from multiple distinct categories of social support, and how these divergent forms work with PCEs to affect emotion dysregulation is complex and contingent on the social support measured.

Additionally, the relationship between emotion dysregulation and social support may be bidirectional or reciprocal (Cohen & Wills, 1985; Gross & John, 2003; Marroquín & Nolen-Hoeksema, 2015; Zaki & Williams, 2013). Indeed, existing literature posits that emotional goals motivate individuals to utilize social supports, or support-seeking behavior, to regulate and cope with their emotions (Gable et al., 2004; Zaki & Williams, 2013). From this perspective, emotion dysregulation may influence the specific social support individuals with ADHD seek and utilize, which in turn impacts their overall emotion dysregulation. Childhood experiences, including PCEs, can influence emotional goals and how individuals evaluate the different domains of social support as effective in regulating their emotions (Collins, 1996; Collins & Read, 1990). Thus, although there is an association between PCEs and social support, individuals’ perceptions of the specific domains of social support may depend on what they deem effective in regulating their emotions. Although they may rate that social support is of high quality, they may not perceive it as effective in helping them regulate their emotions, specifically. Similarly, emotion regulation consists of different domains related to different emotion regulation strategies that also may be essential to identify, to better understand the relationship between the specific social support domains and social support as a whole (Gratz & Roemer, 2004).

Second, the domain of emotion regulation may change the total association between social support and emotion regulation, thereby creating a non-significant analysis. Congruent with this conceptualization of specific domains of social support, further analyses that differentiated the sub-types of social support in the present study told a more nuanced story. Not all types of social support were directly associated with emotion dysregulation. Although associated with PCEs, appraisal social support had no significant relationship with emotion dysregulation outcomes, suggesting that this type of social support may not be associated with emotion regulation outcomes and that it was the presence of self-esteem, tangible, and belonging social support that is protective for emotion regulation outcomes.

Directly, PCEs were not predictors of emotion regulation in adults with ADHD. However, how PCEs were related to emotion regulation was complex. Higher reports of PCEs had relationships with increases in both social support generally and through self-esteem, belonging, and tangible support specifically. Possibly, favorable childhood experiences facilitated social relationships that promoted access to social support later in life.

First, a significant effect was identified in which increased PCEs were related to better emotion regulation indirectly through each self-esteem, belonging, and tangible social support. Alternatively, those with fewer PCEs were associated with lower self-esteem, belonging, and tangible social support and subsequently demonstrated worse emotion regulation. For example, the ability to talk about feelings with family, enjoyment in community traditions, a sense of belonging in high school, and friend support may have had meaningful impacts on an individual. Moreover, the social environment that facilitates PCEs may also have indirectly affected the social environment in which the subsequent adult exists, promoting social support. Similarly, feeling supported by friends, having non-parent adults who cared, and having a supportive family in difficult times may be related to ongoing social support in which childhood environment also influenced the ongoing social environment, in which when faced with challenges, all forms of perceived social support, for their actions or perceptions is a continued pattern. Possibly, this relationship is cyclical, in which those with limited PCEs developed poorer emotion regulation, relying on maladaptive strategies resulting in reduced access to social support, consistent with other studies highlighting the potential bidirectional relationship between social support and emotion regulation (Gross & John, 2003).

Interestingly, although PCEs were associated with appraisal social support, they were not related to emotion regulation outcomes, as appraisal social support had no significant association. Appraisal social support is differentiated from other types of social support such that it involves an individual receiving others’ feedback and guidance to help them evaluate situations, clarify goals, and plan actions (Cohen & Wills, 1985). Individuals with ADHD may solicit, or be offered, opinions and advice for particular situations, confirming or validating coping strategies, even if the strategies used are maladaptive.

Consistent with an IER framework, the utilization of social support to regulate emotion differs and may be contingent on individual perceptions of the benefit of emotion regulation (Williams et al., 2018). Additionally, strategies employed to regulate emotions vary (Gratz & Roemer, 2004; Williams et al., 2018; Zaki & Williams, 2013). When it is believed that social support benefits emotion regulation and is accessed, there are increases in reported well-being (E. O. Cheung et al., 2015; Cohen et al., 1986). Conversely, if an individual with ADHD perceives the received social support as ineffective or invalid, they not only lack the benefits of receiving social support, but may view the interaction as an additional stressor, compounding their coping capacities resulting in poorer emotion regulation, underscoring the importance of perceived social support effectiveness in the context of emotion regulation.

## Limitations

Although the results of the current study demonstrated interesting findings, some limitations must be considered. First, regarding sampling, the current study may be limited due to its use of convenience sampling and a snowball recruitment technique. Studies that utilize a snowball recruitment technique may result in a homogenous sample due to a self-selection bias among participants (Parker et al., 2019). Additionally, the current sample consisted of predominantly White women, with most reporting post-secondary education attainment, which is not representative of the typical ADHD population; therefore, these results must be interpreted with caution. Additionally, participant inclusion was based on a self-reported formal diagnosis or a suspected diagnosis, each of which was corroborated using a standardized measure to assess current ADHD symptoms and longstanding chronicity. However, we did not independently verify self-reports of ADHD diagnoses, which may have compromised diagnostic validity. Next, although analyses were sufficiently powered using a bias-corrected bootstrapped model based on 2,000 bootstrapped samples, the most conservative and robust approach given sample size limitations (Hayes & Scharkow, 2013), repeated research with a larger sample size may better support the current findings with implications for future research directions and understanding of health and well-being for individuals with ADHD. Finally, while the current findings indicate significant indirect effects of PCEs on emotion regulation, it is important to interpret these results with caution. The cross-sectional design in the current study limits the ability to make causal inferences in relation to the temporal ordering of variables, and further, significant indirect effects in cross-sectional data may reflect shared variance among the variables of interest included (Maxwell et al., 2011). The current study highlights identified relationships between PCEs and emotion regulation through social support, but cannot ascertain causality and further research is needed to understand what other factors may impact and interact with identified variables to explain the variance in emotion regulation in adults with ADHD.

## Implications and Conclusions

Overall, there are clear associations between PCEs, social support, and emotion dysregulation in adults with ADHD. Although previous literature individually identified associations between these variables, the current study is the first to assess how social support mediates the relationship between PCEs and emotion regulation capacities in adults with ADHD, attesting to the importance of PCEs and social support. Understanding the importance of PCEs for emotion regulation development, targeted social programs must facilitate ongoing PCEs throughout childhood and foster appropriate social connections for ongoing necessary support. In addition to generally improved health and well-being, improved PCEs and social supports may benefit emotion regulation capacities in adults with ADHD. Further, future research may benefit from expanding these findings and evaluating bidirectional pathways and trajectories of emotion regulation development for individuals with ADHD to improve outcomes into adulthood.
